# Supplementary material for: In vitro performance in cotton plants with different genetic backgrounds: the case of Gossypium hirsutum in Mexico, and its implications for germplasm conservation
Source: PeerJ. 2019 Jun 10;7:e7017. doi: 10.7717/peerj.7017 (PMC6563797; doi:10.7717/peerj.7017)
Supplement: Supplemental Information 1 — Appendix S1.Specifics of the in vitro culture technique used in the establishment and propagation of the germplasm collection. Appendix S2. Scheme of experimental design. Appendix S3. Transgene detection of in vitro germplasm collectio. Appendix S4.In vitro performance at metapopulation level. Appendix S5. Raw sequences from Sanger verification of PCR amplicons. [file peerj-07-7017-s001.docx]

**Supplementary material**

***In vitro* performance in cotton plants with different genetic backgrounds: the case of *Gossypium hirsutum* in Mexico, and its implications for germplasm conservation**

Alejandra Hernández-Terán, Ana Wegier, Mariana Benítez, Rafael Lira, Tania Gabriela Sosa Fuentes, Ana E. Escalante

**Appendix S1.** Specifics of the *in vitro* culture technique used in the establishment and propagation of the germplasm collection

**Disinfection treatment**

Steps

1. Remove the petiole and leaves from the stem of each axillary bud
2. Wash with soap and distilled water all the buds
3. Leave 30 sec in 70% alcohol (96%)
4. Wash with distilled water
5. Leave 10 min in 30% chloride (4%)
6. Wash with distilled water
7. Leave in ethil mercaptian (1 g per 1 l of distilled water) as a fungicide agent till establishment

**Culture medium:** for 1 l of culture medium we used 4.43 g of PhytoTech MS basal medium (PhytoTechnology Laboratories, Shawnee Mission, Kansas, USA solidified with 7 g of Phytagel (Sigma-Aldrich, Darmstadt, Germany) and 30 g of sucrose. Medium pH was adjusted to 5.7 with 0.1 M NaOH prior to addition of the agar. Approximately 6 ml of medium was dispensed into borosilicate culture tubes (25 mm x 95 mm height) and cap-sealed with PhytoTech closures (PhytoTechnology Laboratories, Shawnee Mission, Kansas, USA). Subsequently, each tube was autoclaved at 121ºC and 1.5 kg cm^-2^ for 20 min.

**Propagation technique:** the propagation process was done under sterile conditions in a laminar-flow hood (ThermoFisher, Massachusetts, EUA). To remove the explants from the culture tubes we used forceps and scalpels sterilized into a dry glass bead sterilizer Germinator500 (Stoelting, Illinois, USA).

**Growth and culture rooms: t**he propagation process was done in a specialized culture room with controlled access and a double crystal door to avoid air circulation. The environment of the room was controlled with an air purifier that prevent contamination, additionally, UV irradiation was done in the entire room twice a week. The growth room had environmental controlled conditions (humidity and temperature) and was separated from the culture room by another crystal door. The 12h-photoperiod was provided by cool white fluorescent lamps (Philips, Mumbai, India).

**Appendix S2.** Scheme of experimental design


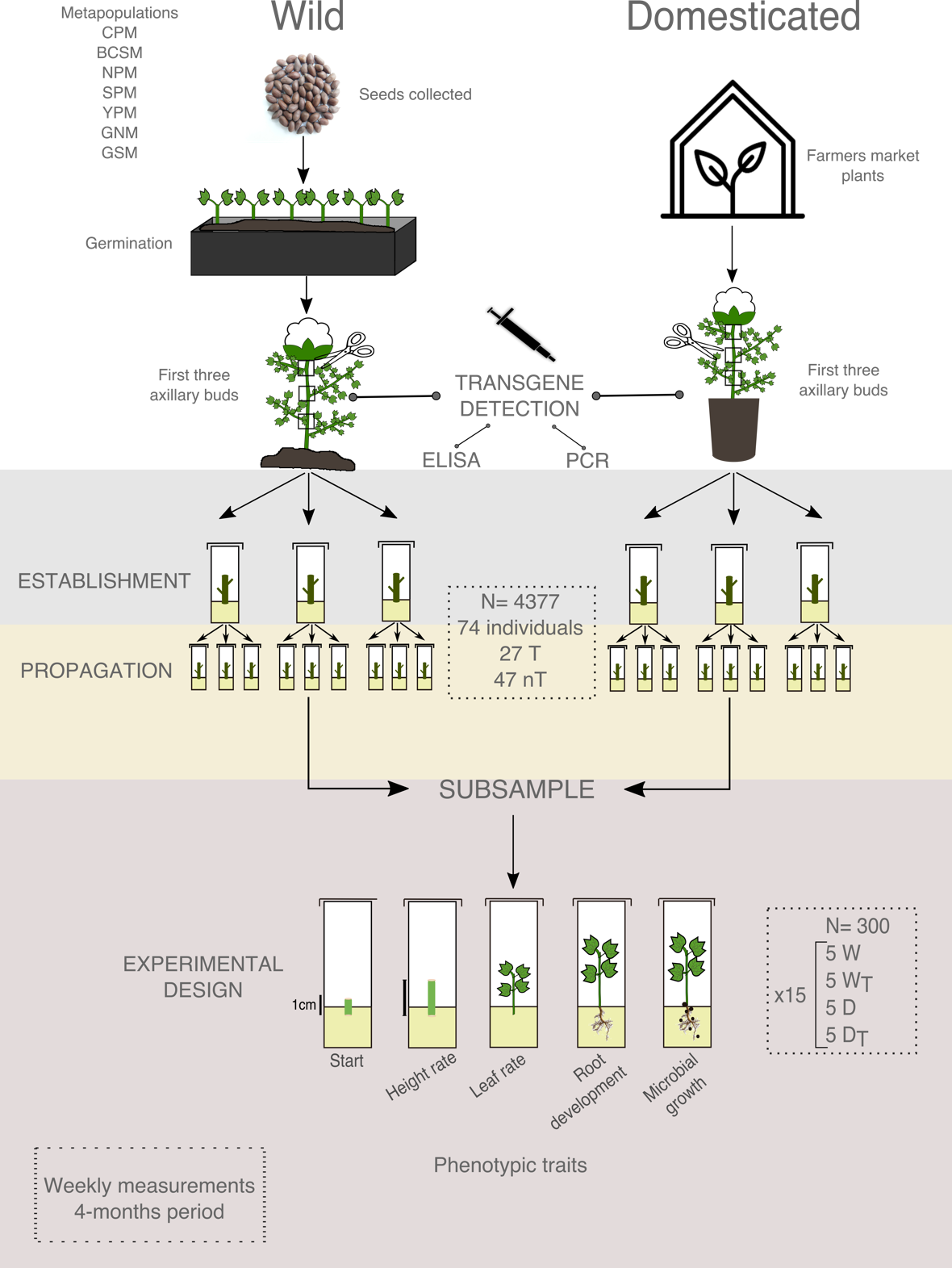


**Figure S2. Scheme of experimental design.** Origin and treatment of the different genotypes (wild and domesticated) of the germplasm collection. In the wild metapopulations collected; CPM: Center Pacific metapopulation, BCSM: Baja California Sur metapopulation, NPM: North Pacific metapopulation, SPM: South Pacific metapopulation, YPM: Yucatan Peninsula metapopulation, GNM: Gulf North metapopulation, GSM: Gulf South metapopulation. In the “propagation” N square= T: transgene presence, nT: without transgene presence. In the “experimental design” N square= W: wild organisms, D: domesticated organisms, W_T_: wild organisms with transgenes and D_T_: domesticated organisms with transgenes.

**Appendix S3.** Transgene detection of *in vitro* germplasm collection

We perform PCR assays for transgene detection in all the individuals from the germplasm collection. We specifically look for some of the transformation events released in Mexico: *Cry1Ab/Ac, Cry2Ab,* and CP4EPSPS (Table S1). The specific PCR conditions for each primer were applied according to the references show in Table S1.

**Table S3. Primers sequence used in the transgene detection protocol.**

| **Primer** | **Sequence** | **Amplicon size** | **Reference** |
| --- | --- | --- | --- |
| *Cry1Ab/Ac* | F 5´ACCGGTTACACTCCCATCGA 3´  R 5´CAGCACCTGGCACGAACT 3´ | 76 bp | Zhang *et al* (2013) |
| *Cry2Ab* | F 5´CAGCGGCGCCAACTCTACG 3´  R 5´TGAACGGCGATGCACCAATGTC 3´ | 260 bp | Randhawa *et al* (2010) |
| CP4 EPSPS | F 5´GCATGCTTCACGGTGCAA 3´  R 5´TGAAGGACCGGTGGGAGAT 3´ | 108 bp | Barbau-Piednoir *et al* (2014) |

**Appendix S4.** *In vitro* performance at metapopulation level


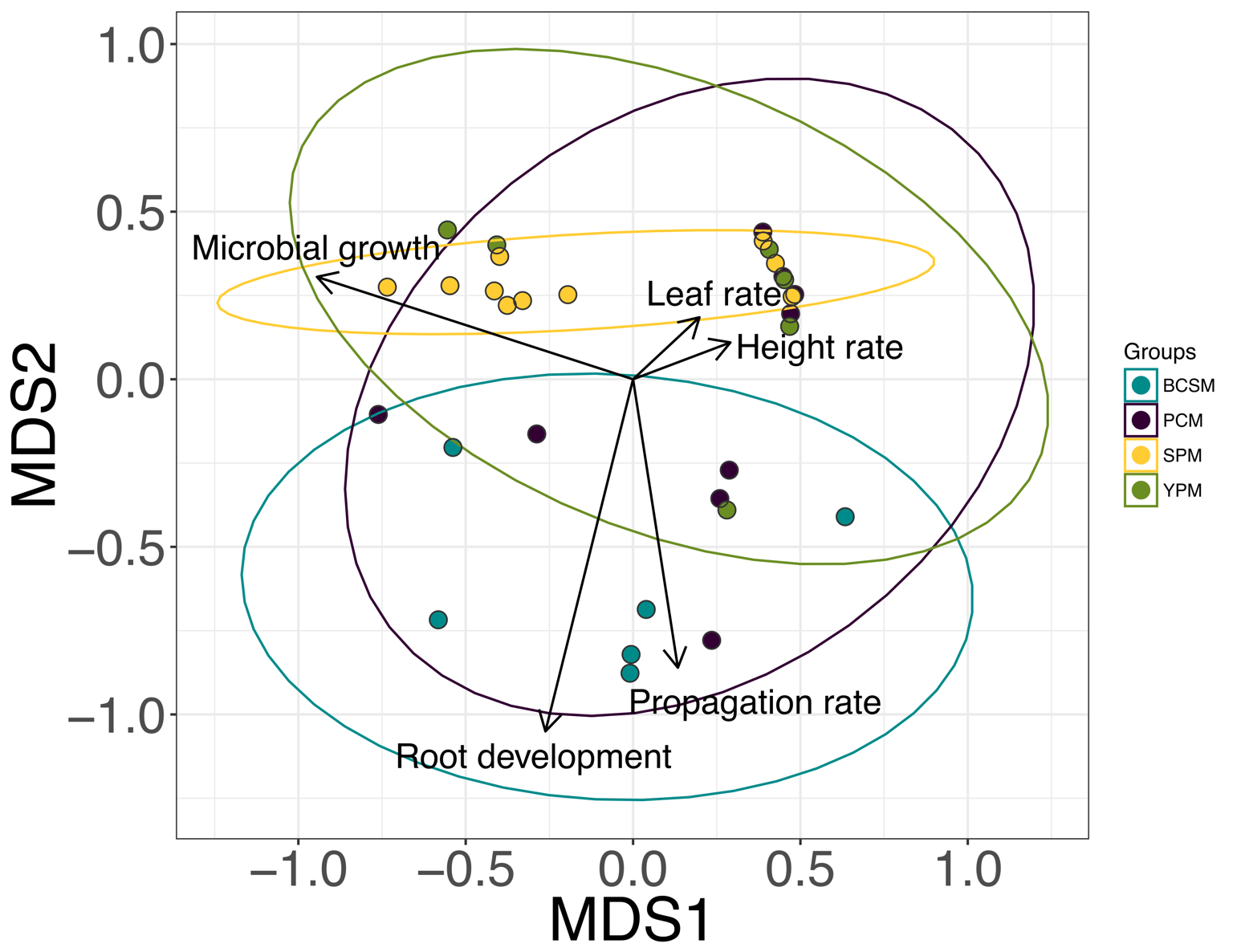


**Figure S4. Non-Metric Multidimensional Scaling that include the analyzed traits in all natural populations without transgene presence.** The ellipses represent 95% confidence interval around the centroids. Populations; BCSM: Baja California Sur metapopulation, CPM: Center Pacific metapopulation, SPM: South Pacific metapopulation and, YPM: Yucatan Peninsula metapopulation. NMDS Stress: 0.16. PERMANOVA *F=* 5.91, *p*= 0.09.

Appendix S5. Integrated analysis of *in vitro* performance between all analyzed genotypes

**Figure S4. Non-Metric Multidimensional Scaling that include the analyzed traits in all analyzed genotypes.** The ellipses represent 95% confidence interval around the centroids. Groups; D= domesticated populations without transgenes, Dt= domesticated populations with transgenes, W= wild populations without transgenes and Wt= wild populations with transgenes. NMDS Stress: 0.151. PERMANOVA *F=* 7.13, *p*= 0.0009.

Appendix S6. Raw sequences from Sanger verification of PCR amplicons

>Seq1 Gossypium hirsutum transgenic insert CP4epsps

AATACTACTCGAAGTCCTCTGGTCTTTCTGGAACCGTCCGTATTCCAGGTGACAAGTCTA

TCTCCCACCGGTCCTTCAAGTTTTGTTTCTTGCTGGTTAGCTGCGTGTAATCGAAAAGGA

CATCGTGCCAACACATTGTGTTGTAATGTTAAAACCACCCTCCTTCCAGCCCTGCTGATC

CTCTTCCCTTAATTCAGTCTTGCGCTGGTTCTCCTGCCAAATCCCGTTTCATTTACAGTA

AAAAATTCAATTCACTTATTCATAAAACAACTTGTCTGGGTCTTTGAACAAACCTCTAAA

CCAGGATGCAACACTGCCATTATACATGTAAGGGTATACAAGTAGTCTTTCGTTAGAAGT

AGCACAATAACCAATCAAACGAAGCAGATTACGGTGAACAGCCAAGCTGATCATCTCCCA

CCGGTCCTTCAAGATTTTTTTTTC

>Seq2 Gossypium hirsutum transgenic insert CP4epsps

AGAAATCTGACTACGTAGTCCTCTGGTCTTTCTGGAACCGTCCGTATTCCAGGTGACAAG

TCTATCTCCCACCGGTCCTTGACTCGTTTCTTTGGTGTCTCCCACCGGTCCTTCAATCGA

TTTAGTGCGGGGGCCTCTTTTATTCCGTTGGCGAGGATAATAAAACCCCCTTTTTGACTG

ATGAGCATATTACCGTACTCTTGTATAACATGGGGCCCTGCCGTGACAATTCCCATACCT

TACTATTAAATACGTATGAAATAAACGCACCCACGACCGTTTTGGGTCTCTTAACTTAGT

CTGAGAATAATAAAGGATGCCATGCGGGGGCAGTGTTCACCTACTTCCCAGTGGTTAGAA

GCGGCCTTAAGTGGGGTAATACCTTGCGTTATTTGTCTCAAACCAATTTGACTATTCCCC

CGGGGCTGCGATTAACTTATTTTTGTGCTGACTAAACTCCAACCCTCACCA

>Seq3 Gossypium hirsutum transgenic insert CP4epsps

GCTACCTGACTCGTAGTCCTCTGGTCTTTCTGGAACCGTCCGTATTCCAGGTGACAAGTC

TATCTCCCACCGGTCCTTCAACGGTGGTTGTTGGTGAGAGGGTTGAATTGTGATTATTAC

CGACGGCCTTTTATTATCATTTTCTTAGCGTTAACACCCTACAATTTGATGGAAGCGGCT

CGACATGACTTATGTACAAGGCCCTCGAGCATACAATGCCCCCCACCACAGGAAAATATA

CGAGATTTTGCCTACTCGTGACCACTTCAAATTATAAAAATTAGATAGAGACGTGAAAAA

AGGGTTTTAATTTTTCCACCCCCCAATTGATTCTAAGAAACAATGGGCGGGCCTCTGGAG

TATAACCCTGAT

>Seq4 Gossypium hirsutum transgenic insert CP4epsps

AAAACTCTCGTAGTCCTCTGGTCTTTCTGGACCGTCCGATATTCCAGGTGACAAGTCTAT

CTCCCACCGGTCCTTCAATCTGTTATTGGTGAACCCATTGTTAAGGAAAATCACCGAGGT

GCCTTACTTCCCTTTATTGTTATTATAAACTCATTCGTCCCTGCTTAGAAACCAAAATAA

TGAATAAACAACGAGCTGCAAAAACCTAATCCATCCAATGCTCTTAAAAGATGGAGAGTA

TAACCACCCACTTTTCTCTCCTTAATATAAAACAATAATGAAAAAAGTTGTAAAAGCATG

TTCTTGTTTGATCCCTTCTTAATGATTATAAGAAAAAAAAAGTCATCTAAGTATGAATAT

TACTGTTTC

>Seq5 Gossypium hirsutum transgenic insert Cry1ab

GGGGTGCGTGAATCACGAGATCGAGAACAACACCGACGAGCTTAAGTTCTCCAACTGCGT

CGAGGAAGAAATCTATCCCAACAACACCGTTACTTGCAACGACTACACTGTGAATCAGGA

AGAGTACGGAGGTGCCTACACTAGCCGTAACAGAGGTTACAACGAAGCTCCTTCCGGTCC

TGCTGACTATGCCTCCGTCCTCTTCCTGATTCACAGTGTAGTCGTTGCAAGTAACGGTGT

TGTTGGGATATAATTTCCTTCCTCGACACAGTTGGAGAACTTAAGCTCGTCGGTTTTGTT

CTCGATGGCGTGGATGGTCACGCAACCCTCATACCCTCCTTGTAGGCGGTCAAA

>Seq6 Gossypium hirsutum transgenic insert Cry1ab/ac

CTCGCGCTTACTGCCTCAGTCGAGATCGAGAACAACACCGACGAGCTTAAGTTCTCCAAC

TGCGTCGAGGAAGAAATCTATCCCAACAACACCGTTACTTGCAACGACTACACTGTGAAT

CAGGAAGAGTACGGAGGTGCCTACACTAGCCGTAACAGAGGTTACAACGAAGCTCCTTCC

GGTCCTGCTGACTATGCCTCCGTCATCTTCCTGATTTCAGTGTGGTCGCTTGCAAGTAAC

GGTTTTGTTGGGATAGATTTCTTCCTCGACGCAGTTGGAGAACTTAAGCTCGTCGGTGTT

GTTCTCGATGGGGTGGATGGTCACGCAACCCTCCTACCCTCCTTGTAGGCGGTCCAA

>Seq7 Gossypium hirsutum transgenic insert Cry1ab/ac

GGATTGCACTCTGAGTAGCGAGATCGAGACGCACCGACGAGCGTTAAGTTCTCCAACTGC

GTCGAGGAAGAAATCTATCCCAACAACACCGTTACTTGCAACGACTACACTGTGAATCAG

GAAGAGTACGGAGGTGCCTACACTAGCCGTAACAGAGGTTACAACGAAGCTCCTTCCGGT

CCTGCTGACTATGCCTCCGTAAAACATCCTGATCCCCCCTGCACCCCCTGCAACAACCGG

TGTTGTTGGGATAGATTACTTCCTCGACCCAGCCGGATAACTTATACTCGTACTTGTTGT

TTGTGATGACGAGGAATACCTACCTACTTACCACTCCGTGCCTTGAATTAACGCTAATTG

ATGAGAGACCATTCGAGCCCATGACAATAACCCTCTCTTGTCTTATCCTTTTTTTGTTAT

ACCTCTCCCCCCCG

>Seq8 Gossypium hirsutum transgenic insert Cry1ab/ac

GAGCGCTCTGCAGAGTCCGGTTCGTGACAACACCGACGAGCTTAAGTTCTCCAACTGCGT

CGAGGAAGAAATCTATCCCAACAACACCGTTACTTGCAACGACTACACTGTGAATCAGGA

AGAGTACGGAGGTGCCTACACTAGCCGTAACAGAGGTTACAACGAAGCTCCTTCCGGTCC

TGCTGACTATGCCTCCGTACTCTTCCTGATATTCAGTGTGTACGTTTGCGGTAACGGTGT

TGTTGGGATAGATTTCTTCCTCGACGCACTTGGAGAACTTAAGCTCGTCGGTTTTGTTCT

CGATGTCGTGGATGGTCACGCCCCCCGACCCCCCCGGGGGGGGGGGAAAAAAAAC

>Seq9 Gossypium hirsutum transgenic insert Cry1ab/ac

GACACTGCGTGAATCACGAGATCGAAAACAACACCGACGAGCTTAAGTTCTCCAACTGCG

TCGAGGAAGAAATCTATCCCAACAACACCGTTACTTGCAACGACTACACTGTGAATCAGG

AAGAGTACGGAGGTGCCTACACTAGCCGTAACAGAGGTTACAACGAAGCTCCTTCCGGTC

CTGCTGACTATGCCTCCGTCATCTTCCTGCTTGCGGTGTCGTTGGTGGCACGGGTTGGTT

TGGGTGGATTTTATCCCTCCCTCGATTTGGAGAAGATAAGCTACGCTGGTTTGTTTTGTT

TTCGATGGAGTGGATAGTCACGCATCCCCTGTAACCCTTCCTGTAGGCGGTCAAAAA

>Seq10 Gossypium hirsutum transgenic insert Cry1ab/ac

GACACTGCGTGAATCACGAGATCGAAAACAACACCGACGAGCTTAAGTTCTCCAACTGCG

TCGAGGAAGAAATCTATCCCAACAACACCGTTACTTGCAACGACTACACTGTGAATCAGG

AAGAGTACGGAGGTGCCCACACTAGCCGTAACAGAGGTTACAACGAAGCTCCTTCCGGTC

CTGCTGACTATGCCTCCGTCATCTTCCTGCTTACGGTGTCGTTGGTGGCACGGGTTGGTT

TGGGTGGATTTTATCCCTCCCTCGAGTTGGAGAAGATAAGCTACGCTGGTTTGTTTTGTT

TTCGATGGAGTGGATAGTCACGCATCCCCTGTAACCCTTCCTGTAGGCGGTCAAAAA

>Seq11 Gossypium hirsutum transgenic insert CP4epsps

AATACTACTCGAAGTCCTCTGGTCTTTCTGGAACCGTCCGTATTCCAGGTGACAAGTCTA

TCTCCCACCGGTCCTTCAAGTTTTGTTTCTTGCTGGTTAGCTGCGTGTAATCGAAAAGGA

CATCGTGCCAACACATTGGGTTGTAATGTTAAAACCACCCTCCTTCCAGCCCTGCTGATC

CTCTTCCCTTAATTCAGTCTTGCGCTGGTTCTCCTGCCAAATCCCGTTTCATTTACAGTA

AAAAATTCAATTCACTTATTCATAAAACAACTTGTCTGGGTCTTTGAACAAACCTCTAAA

CCAGGATGCAACACTGCCATTATACATGTAAGGGTATACCAGTAGTCTTTCGTTAGAAGT

AGCACAATAACCAATCAAACGAAGCAGATTACGGTGAACAGCCAAGCTGATCATCTCCCA

CCGGTCCTTCAAGATTTTTATTTC

>Seq12 Gossypium hirsutum transgenic insert CP4epsps

AGAAATCTGACTACGTAGTCCTCTGGTCTTTCTGGAACCGTCCGTATTCCAGGTGACAAG

TCTATCTCCCACCGGTCCTTGACTCGTTTCTTTGGTGTCTCCCACCGGTCCTTCAATCGA

TTTAGTGCGGGGGCCTCTTTTATTCCGTTGGCGAGGATAATAAAACCCCCTTTTTGACTG

ATGAGCATATTACCGTACTCTTGTATAACATGGGGCCCTGCCGTGACAATTCCCATACCT

TACTATTAAATACGTATAAAATAAACGCACCCACGACCGTCTTGGGTCTCTTAACTTAGT

CTGAGAATAATAAAGGATGCCATGCGGGGGCAGTGTTCACCTACTTCCCAGTGGTTAGAA

GCGGCCTTAAGTGGGGTAATACCTTGCGTTATTTGTCTCTAACCAATTTGACTATTCCCC

CGGGGCTGCGATTACCTTATTTTTGTGCTGACTAAACTCCAACCCTCACCA

>Seq13 Gossypium hirsutum transgenic insert CP4epsps

GCTACCTGACTCGTAGTCCTCTGGTCTTTCTGGAACCGTCCGTATTCCAGGTGACAAGTC

TATCTCCCACCGGTCCTTCAACGGTGGTTGTTGGTGAGAGGGTTGAATTGTGATTATTAC

CGACGGCCTTTTATTATCAATTTCTTAGCGTTAACACCCTACAATTTGATGGAAGCGGCT

CGACATGACTTATGTACAAGGCCCTCGAGCATCCAATGCCCCCCACCACAGGAAAATATA

CGAGATTTTGCCTACTCGTGACCACTCCAAATTATAAAAATTAGATAGAGACGTGAAAAA

AGGGTTTTAATTTTTCCACCCCCCAATTGATTCTAAGAAACAATGGGCGGGCCTCTGGAG

TATAACCCTGATT

>Seq14 Gossypium hirsutum transgenic insert CP4epsps

AAAACTCTCGTAGTCCTCTGGTCTTTCTGGACCGTCCGATATTCCAGGTGACAAGTCTAT

CTCCCACCGGTCCTTCAATCTGTTATTGGTGAACCCATTGTTAAGGAAAATCACCGAGGT

GCCTTACTTCCCTTTATTGTTATTATAAACTCATTCGTCCCTGCTTAGAAACCAAAATAA

TGAATAAACAACGAGCTGCAAAAACCTAATCCATCCAATGCTCTTAAAAGATGGAGAGTA

TAACCACCCACTTTTCTCTCCTTAATATAAGACAATAATGAAAAAAGTTGTAAAAGCATG

TTCTTGTTTGATCCCTTCTTAATGATTATAAGAAAACTAGACAGTCATCTAAGTATGAATAT

TACTGTTTC

>Seq15 Gossypium hirsutum transgenic insert Cry1ab/ac

GGGGTGCGTGAATCACGAGATCGAGAACAACACCGACGAGCTTAAGTTCTCCAACTGCGT

CGAGGAAGAAATCTATCCCAACAACACCGTTACTTGCAACGACTACACTGTGAATCAGGA

AGAGTAGGGAGGTGCCTACACTAGCCGTAACAGAGGTTACAACGAAGCTGCTTCCGGTCC

TGCTGACTATGCCTCCGTCCTCTTCTTGATTCACAGTGTAGTCGTTGCAAGTAACGGTGT

TGTTGGGATATAAATTCCTTCCTCCACACAGTTGGAGAACTTAAGCTCGTCGGTTTTGTT

CTCGATGGCGTGGATGGTCACGCAACCCTCATACCCTCCTTGTAGGCGGTCAAA

>Seq16 Gossypium hirsutum transgenic insert Cry1ab/ac

CTCGCGCTTACTGCCTCAGTCGAGATCGAGAACAACACCGACGAGCTTAAGTTCTCCAAC

TGCGTCGAGGAAGAAATCTATCCCAACAACACCGTTACTTGCAACGACTACACTGTGAAT

CAGGAAGAGTACGGAGGTGCCTACACTAGCCGTAACAGAGGTTACAACGAAGCTGCTTCC

GGTCCTGCTGACTATGCCTCCGTCATCTTCCTGATTACAGTGTGGTCGCTTGCAAGTAAC

GGCTCTGTTGGGATAGATTTTTTCCTCGACGCAGTTGGAGAACTTAAGCTCGTCGGTGTT

GTTCTCGATGGGGTGGATGGTCACGCAACCCTCCTACCCTCCTTGTAGGCGGTCCAA

>Seq17 Gossypium hirsutum transgenic insert Cry1ab/ac

GGATTGCACTCTGAGTAGCGAGATCGAGACGCACCGACGAGCGTTAAGTTCTCCAACTGC

GTCGAGGAAGAAATCTATCCCAACAACACCGTTACTTGCAACGACTACACTGTGAATCAG

GAAGAGTACGGAGGTGCCTACACTAGCCGTAACAGAGGTTACATCGAAGCTCCTTCCGGT

CCTGCTCACTATGCCTCCGTAAAACATCCTGATCCCCCCTGCACCCCCTGCAACAACCGG

TGTTGTTGGGATAGATTACTTCCTCGACCCAGCCGGATAACTTATACTCGTACTTGTTGT

TTGTGATGACGAGGAATACCTACCTACTTTACCACTCCGTGCCTTGAATTAACGCTAATTG

ATGAGAGACCATTCGAGCCCATGACAATAACCCTCTCTTGTCTTATCCTTTTTTTGTTAT

ACCTCTCCCCCCCG

>Seq18 Gossypium hirsutum transgenic insert Cry1ab/ac

GAGCGCTCTGCAGAGTCCGGTTCGTGACAACACCGACGAGCTTAAGTTCTCCAACTGCGT

CGAGGAAGAAATCTATCCCAACAACACCGTTACTTGCAACGACTACACTGTGAATCAGGA

AGAGTACGGAGGTGCCTACACTAGCCGTAACAGAGGTTACAACGAAGCTCCTTCCGGTCC

TGCTGACTATGCCTCCGTACTCTTCCTGATATTCAGTGTGTACGTTTGCGGTACCGGTGT

TGTTGGGATAGATTTCTTCCTCGACGCACTTGGAGAACTTAAGCTCGTCGGTTTTGTTCT

CGATGTCGTGGATGATCACGCCCCCCGACCCCCCCGGGCGGGGGGGAAAAAAATC

>Seq19 Gossypium hirsutum transgenic insert Cry1ab/ac

GACACTGCGTGAATCACGAGATCGAAAACAACACCGACGAGCTTAAGTTCTCCAACTGCG

TCGAGGAAGAAATCTATCCCAACAACACCGTTACTTGCAACGACTACACTGTGAATCAGG

AAGAGTACGGAGGTGCCTACACTAGCCGTAACAGACGTTACAACGAAGCTCCTTCCGGTC

CTGCTGACTATGCCTCCGTCATCTTCCTGCTTGCGGTGTCGTCGGTGGCACGGGTTGGTT

TGGGTGGATTTTATCTCTCCCTCGATTTGGAGAAGATAAGCTCCGCTGGTTTGTTTTGTT

TTCGATGGAGTGGATAGTCACGCATCCCCTGTAACCCTTCCTATAGGCGGTCAAAAA

>Seq20 Gossypium hirsutum transgenic insert Cry1ab/ac

GTAAACTCTGAGTGAGCGTTATCGTCCGATACACCGACGAGCTTAAGTTCTCCAACTGCG

TCGAGGAAGAAATCTATCCCAACAACACCGTTACTTGCAACGACTACACTGTGAATCAGG

AAGAGTACGGAGGTGCCTACATTAGCCGTAACAGAGGTTACAACGAAGCTCGTTCCGGTC

CTGCTGACTATGCCTCCGTAACTCTTCCTGATTCACAGTGTAGTCGTTGCAAGTAACGGT

GTTGTTGGGATAGATTTCTTCCTCCACGCCTTTGGAGAACTTAAGCTCGTCGGTGTTGTT

CTCGATCTCGTGGATGGTCACGCAAC

>Seq21 Gossypium hirsutum transgenic insert CP4epsps

AATACTACTCGAAGTCCTCTGGTCTTTCTGGAACCGTCCGTATTCCAGGTGACAAGTCTA

TCTCCCACCGGTCCTTCAAGTTTTGTTTCTTGCTGGTTAGCTGCGTGTAATCGAAAAGGA

CATCGAGCCAACACATTGTGTTGTAATGTTAAAACCACCCTCCTTCCAGCCCTGCTGATC

CTCTTCCCTTAATTCAGTCTTGCGCTGGTTCTCCTGCCAAATTCCGTTTCATTTACAGTA

AAAAATTCAATTCACTTATTCATAAAACAACTTGTCTGGGTCTTTGAACAAACCTCTAAA

CCAGCATGCAACACTGCCGTTATACATGTAAGGGTATACAAGTAGTCTTTCGTTAGAAGT

AGCACAATAACCAATCAAACGAAGCAGATTACGGTGAACAGCCAAGCTGATCATCTCCCA

CCGGTCCTTCAAGATTTTTTTTTC

>Seq22 Gossypium hirsutum transgenic insert Cry1ab/ac

GACACTGCGTGAATCACGAGATCGAAAACAACACCGACGAGCTTAAGTTCTCCAACTGCG

TCGAGCAAGAAATCTATCCCAACAACACCGTTACTTGCAACGACTACACTGTGAATCAGG

AAGAGTACGGAGGTGCCTACACTAGCCGTAACAGAGGTTACAACGAAGCTCCTCCCGGTC

CTGCTGACTATGCCTCCGTCATCTTCCTGCTTGCGGTGTCGTTGGTGGCACGGGTTGGTT

TGGGAGGATTTTATCCCGCCCTCGATTTGGAGAAGATAAGCTACGCTGGTTTGTTTTGTT

TTCGATGGAGTGGATAGTCACGCATCCCCTGTAACCCTTCCTGTAGGCGGTCAAAAA

>Seq23 Gossypium hirsutum transgenic insert CP4epsps

AAAACTCTCGTAGTCCTCTGGTCTTTCTGGACCGTCCGATATTCCAGGTGACAAGTCTAT

CTCCCACCGGTCCTTCAATCTGTTATTGGTGAACCCATTGTTAAGGAAAATCACCGAGGT

GCCTTACTTCCCTTTATTGTTATTATAAACTCATTCGTCCCTGCTTAGAAAACAAAATAA

TGAATAAACAACGAGCTGCAAAAACCGAATCCATCCAATGCTCTTAAAAGATGGAGAGTA

TAACCACCCACTTTTCTCTCCTTAATCTAAGACAATAATGAAAAAAGTTGTAAAAGCATG

TTCTTGTTTGATCCCTTCTTAATGATGATAAGCAAACTAGACAGTCATCTAAGTATGAATAT

TACTGTTTC

>Seq24 Gossypium hirsutum transgenic insert Cry1ab/ac

GTAAACTCTGAGTGAGCGTTATCGTCCGATACACCGACGAGCTTAAGTTCTCCAACTGCG

TCGAGGAAGACATCTATCCCAACAACACCGTTACTTGCAACGACTACACTGTGATTCAGG

AAGAGTGCGGAGGTGCCTACACTAGCTGTAACAGAGGTTACAACGAAGCTCCTTCCGGTC

CTGCTGACTATGCCTCCGTAACTCTTCCTGACTCACAGTGTAGTCGTTGCAAGTAACGGT

GTTGTTGGGATAGATTTCTTCCTCGACGCATTTGGAGAACTTAAGCTCGTCGGTGTTGTT

CTCGATCTCGTGGATGGCCACGCAAC

>Seq25 Gossypium hirsutum transgenic insert Cry1ab/ac

GGATTGCACTCTGAGTAGCGAGATCGAGACGCACCGACGAGCGTTAAGTTCTCCAACTGC

GTCGAGGAAGAAATCTATCCCAACAACACCGTTACTTGCAACGGCTACACTGTGAATCAG

GAAGAGTGCGGAGGTGCCTACACTAGCCGTAACAGAGGTTACAACGAAGCTCCTTCCGGT

CCTGCTGACTATGCCTCCGTAAAACATCCTGATCCCCCCTGCACCCCCTGCAACAACCGG

TGTTGTTGGGATAGATTACTTTCTCGACCCAGCCGGATAACTTATACTCGTACTTGTTGT

TTGTGATGACGAGGAATACCTACCTACTTACCACTCCGTCCCTTTAATTAACGCTAATTG

ATGAGAGACCATTCGAGCCCATGACAATAACCCTCTCTTGTCTTATCCTTTTTTTGTTAT

ACCTCTCCCCCCCG

>Seq26 Gossypium hirsutum transgenic insert CP4epsps

AAAACTCTCGTAGTCCTCTGGTCTTTCTGGACCGTCCGATATTCCAGGTGACAAGTCTAT

CTCCCACCGGTCCTTCAATCTGTTATTGGTGAACCCATTGTTAAGGAAAATCACCGAGGT

GCCTTACTTCCCTTTATTGTTATTATAAACTCATTCGTCCCTGCTTAGAAACCAAAATAA

TGAATAAACAACGAGCTGCAAAAACCTAATCCATCCAATGCTCTGAAAAGATGGAGAGTA

TAACCACCCACTTTTCTCTCCTTAATATAAAACAATAATGAAAAAAGTTGTAAAAGCATG

TTCTTGTTTGATCCCTTCTTAATGATTATAAGAACATAAAAGTCATCTAAGTATGAATAT

TACTGTTTC

>Seq27 Gossypium hirsutum transgenic insert Cry1ab/ac

GACACTGCGTGAATCACGAGATCGAAAACAACACCGACGAGCTTAAGTTCTCCAACTGCG

TCGAGAAAGAAATCTATCCCAACAACACCGTTACTTGCAACGACTACACTGTGAATCAGG

AAGAGTACGGAGGTGCCTACACTAGCCGTAACAGAGGTTACAACGAAGCTCCTTCCGGTC

CTGCTGACTATCCCTCCGTCATCTGCCTGCTTGCGGTGTCGTTGGTGGCACGGGTTGGTT

TGGGTGGATTTTATCCCTCCCTCGATTTGGAGAAGATAAGCTACGCTGGTTTGTTTTGTT

TTCGATGGAGTGTATAGTCACGCATCCCCTGTAACCCTTCCTGTAGGCGGTCTACAG
